# Molecular and archaeological evidence on the geographical origin of domestication for *Camelina sativa*

**DOI:** 10.1002/ajb2.16027

**Published:** 2022-07-11

**Authors:** Jordan R. Brock, Melissa M. Ritchey, Kenneth M. Olsen

**Affiliations:** ^1^ Department of Biology Washington University in St. Louis St. Louis Missouri 63130 USA; ^2^ Department of Horticulture Michigan State University East Lansing Michigan 48824 USA; ^3^ Department of Anthropology Washington University in St. Louis St. Louis Missouri 63130 USA

**Keywords:** allopolyploid, biofuel, Brassicaceae, *Camelina*, crop mimicry, domestication, population genetics, weed, wild crop relatives

## Abstract

**Premise:**

Camelina (gold‐of‐pleasure or false flax) is an ancient oilseed crop with emerging applications in the production of sustainable, low‐input biofuels. Previous domestication hypotheses suggested a European or western Asian origin, yet little genetic evidence has existed to assess the geographical origin for this crop, and archaeological data have not been systematically surveyed.

**Methods:**

We utilized genotyping‐by‐sequencing of 185 accessions of *C. sativa* and its wild relatives to examine population structure within the crop species and its relationship to populations of its wild progenitor, *C. microcarpa*; cytotype variation was also assessed in both species. In a complementary analysis, we surveyed the archaeological literature to identify sites with archaeobotanical camelina remains and assess the timing and prevalence of usage across Europe and western Asia.

**Results:**

The majority of *C. microcarpa* sampled in Europe and the United States belongs to a variant cytotype (2n = 38) with a distinct evolutionary origin from that of the crop lineage (2n = 40). Populations of *C. microcarpa* from Transcaucasia (South Caucasus) are most closely related to *C. sativa* based on cytotype and population structure; in combination with archaeological insights, these data refute prior hypotheses of a European domestication origin.

**Conclusions:**

Our findings support a Caucasus, potentially Armenian, origin of *C. sativa* domestication. We cannot definitively determine whether *C. sativa* was intentionally targeted for domestication in its own right or instead arose secondarily through selection for agricultural traits in weedy *C. sativa*, as originally proposed by Vavilov for this species.

*Camelina sativa* (L.) Crantz (also known as camelina, false‐flax, and gold‐of‐pleasure) is an oilseed crop in the Brassicaceae used as a biofuel and in a broad range of other applications. Camelina seeds contain high concentrations of oil (30–47% by dry weight), with a high ratio of unsaturated fatty acids and a fatty acid content amenable for use as an aviation biofuel and biodiesel (Gunstone and Harwood, 2007; Moser, 2010). Residual protein‐rich seed meal is suitable for animal feed, with low glucosinolate content and high levels of omega‐3 fatty acids (Pilgeram, 2007; Berhow et al., 2013). Camelina's close phylogenetic relationship to the well‐studied model system *Arabidopsis thaliana* (L.) Heynh (Beilstein et al., 2006, 2008) enables a wide range of opportunities for crop improvement, genome editing, and evolutionary studies. Further, its short generation time (Gugel and Falk, 2006), ability to grow on marginal and saline soils (Moser, 2010), disease resistance (Séguin‐Swartz et al., 2009), and ease of genetic transformation (Lu and Kang, 2008) leave camelina well positioned for future applications in agriculture. The existence of winter and spring annual crop types provides flexibility in agricultural applications, as the crop can be grown as a winter cover crop or a short‐cycle spring crop. Camelina's low input requirements and high yields combined with its increased disease and pest resistance relative to canola (Séguin‐Swartz et al., 2009; Eynck et al., 2012; Soroka et al., 2015) make it an appealing oilseed crop alternative. Beyond its applied agricultural value, the growing set of genetic and genomic tools for camelina, including a reference genome (Kagale et al., 2014) and developmental transcriptome atlas (Kagale et al., 2016), provides a framework for leveraging this species as a model organism for evolutionary and molecular studies.

Camelina has been studied by researchers worldwide for over a century, with an early focus on agricultural and weedy variants of the species as a model system for genetics, plasticity, and crop mimicry in plants (Zinger, 1909; Tedin, 1925; Sinskaya and Beztuzheva, 1931). The species’ high degree of phenotypic plasticity in growth habit and its ability to vegetatively mimic flax plants in crop fields was extensively studied as a mechanism that facilitated its adaptation as an agricultural weed (Barrett, 1983). Nikolai Vavilov, the pioneer of crop domestication research, considered *C. sativa* a “secondary crop,” where selection for agricultural traits first occurred during its adaptation as a cryptic weed of flax fields, only later to be co‐opted as an intentionally cultivated crop in its own right (Vavilov, 1926).

Several traits were targets of selection during *C. sativa*'s domestication, including increased seed fruit and seed size, loss of shattering, and loss of vernalization requirements in spring varieties (Figure 1). Archaeological evidence suggests that its small‐seeded wild progenitor species, *C. microcarpa* Andrz. ex DC, was likely collected or cultivated intentionally before the emergence of recognizable domestication traits (Hovsepyan and Willcox, 2008). Evidence of domesticated *C. sativa* cultivation in Europe arises only toward the end of the Bronze Age and early Iron Age (~1200 BCE) (Knörzer, 1978; Zohary et al., 2012). Over the course of the Iron Age, camelina cultivation became more prevalent throughout Europe (Van der Veen et al., 2008; Zohary et al., 2012; Larsson, 2013), where it was used for food and fuel. It remained an important oilseed crop across Europe and western Asia until the mid‐20th century, after which cultivation was largely abandoned in favor of oilseed rape and other higher‐yielding oilseeds.

**Figure 1:**
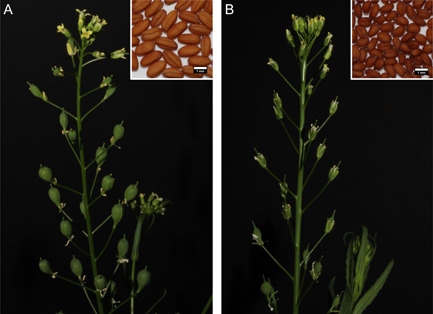
Growth habit of (A) *Camelina sativa* and (B) *C. microcarpa* (2n = 40). Rapidly developing and inflated seed pods and substantially larger seeds can be observed in the domesticate in comparison to *C. microcarpa*. Scale bars = 1 mm.

Recent population genetic studies of *C. sativa* have revealed low to modest genetic diversity in modern cultivars (Vollmann et al., 2005; Ghamkhar et al., 2010; Luo et al., 2019; Li et al., 2021). This dearth of diversity is likely largely attributable to the loss of landrace and cultivar diversity following 20th‐century declines in camelina cultivation. Abandonment of camelina farming is also almost certainly a key factor in the near disappearance of the species as an agricultural weed and the apparent absence of feral populations in modern times (Brock et al., 2018); this decline is strikingly evident in the precipitous drop in *C. sativa* herbarium collections made after the mid‐20th century (Martin et al., 2017). Additional contributing factors in the decline of weedy and feral camelina populations likely include improved techniques for removing weed seeds in flax harvests (Mirek, 1997) and increased herbicide use in European agriculture.

The geographical origin of domestication for *C. sativa* has remained ambiguous. A previous study utilized AFLPs to characterize diversity within *C. sativa* and concluded that the Russia‐Ukraine region of Eastern Europe is the likely center of origin for the crop (Ghamkhar et al., 2010). Another study using a single‐nucleotide polymorphism (SNP) array failed to determine a geographical origin for *C. sativa*, and found no clear geographic clustering of extant *C. sativa* varieties (Singh et al., 2015). Considering that many, if not most, unique cultivars of *C. sativa* were likely lost in the 20th century, it may be difficult to infer a geographical origin of domestication based on present‐day regions of high cultivar diversity. To the best of our knowledge, no attempt has been made previously to utilize populations of the wild progenitor, *C. microcarpa*, to provide insights on the geographical origin of camelina domestication.

*Camelina sativa* is an allohexaploid species (2n = 6x = 40), and only recently have studies uncovered its phylogenetic relationships to wild *Camelina* species (Brock et al., 2018; Mandáková et al., 2019; Žerdoner Čalasan et al., 2019; Chaudhary et al., 2020). Using genome‐wide SNP markers, Brock et al. (2018) determined phylogenetic relationships in the genus and provided genetic evidence that *C. sativa* is derived from the wild hexaploid species *C. microcarpa*. In complementary work, Mandáková et al. (2019) utilized comparative chromosome painting and genomic in situ hybridization to elucidate a nearly identical chromosome structure and common macroevolutionary origin for the subgenomes of *C. sativa* and *C. microcarpa* (Mandáková et al., 2019). A more recent study has determined that the timing of divergence of the chloroplast genomes of domesticated *C. sativa* and wild *C. microcarpa* is consistent with the origins of agriculture 10–12 Kya (Brock et al., 2022). For the wild progenitor species, genotyping‐by‐sequencing of geographically widespread populations has revealed two highly genetically diverged subgroups (*F*_ST_ ~0.3) that appear to be reproductively isolated in the wild (Brock et al., 2020); cytotype characterizations indicate that these within‐species subgroups correspond to two distinct cytotypes: 2n = 6x = 40 (the cytotype shared with domesticated *C. sativa*) and 2n = 6x = 38 (Chaudhary et al., 2020; Brock et al., 2022). Recent chromosome characterization of the two *C. microcarpa* cytotype variants further indicates that they evolved through independent allopolyploidization events in the late Pleistocene, with only two of their three subgenomes shared between them (Brock et al., 2022).

In this study we combined genome‐wide sequencing analyses of camelina and its wild progenitor with an analysis of archaeobotanical data to assess the domestication history of this reemerging oilseed crop. Our specific aims were to (1) determine the likely geographic center of domestication of *C. sativa*; and (2) identify geographical distributions of the two evolutionarily diverged cytotype groups within the wild progenitor, *C. microcarpa*, and the roles of each in camelina domestication. Our findings on camelina's domestication history and present‐day distribution of reproductively compatible wild populations can inform strategies for effective agricultural practices in this resurgent oilseed crop.

## MATERIALS AND METHODS

### Collections

Previously reported collections of *Camelina* from Turkey, Georgia, Armenia, Ukraine, and the United States, and from the USDA GRIN germplasm collection, were utilized for genotyping‐by‐sequencing (GBS) analyses (Brock et al., 2018, 2020). Taxa included *C. sativa*, *C. microcarpa* (2n = 38 and 2n = 40 cytotypes), *C. rumelica*, and *C. neglecta*. Among the *C. sativa* individuals included in the following analyses, only three (Ames 31231, Ames 31232, and JRB 153) could be definitively considered as collected from wild or weedy contexts. Two other accessions (PI 633192 and PI 633194) from the USDA GRIN germplasm collection were labeled as wild, although there is no additional evidence to support this designation. GPS coordinates and approximate localities for collections can be found in Appendix S1.

### Genotyping‐by‐sequencing

Preparation of DNA and sequencing libraries was conducted as in Brock et al. (2020) and is summarized as follows: DNA was extracted from leaf material collected from single individuals belonging to 185 accessions, using either a modified CTAB DNA extraction protocol (Brock et al., 2018) or DNeasy Plant Mini Kits (Qiagen, Valencia, California, USA). Genotyping‐by‐sequencing libraries were then prepared with a method modified from Elshire et al. (2011) as follows: digestion of 100 ng gDNA was performed with 0.4 µL of NEB ApeKI restriction enzyme (New England Biolabs, Ipswich, Massachusetts, USA) and incubation at 75°C for 2 h. Ligation of adapters was conducted on digested DNA with NEB T4 ligase at 22°C for 2 h then 65°C for 20 min. Cleanup of the ligation was performed using 25 µL AMPure XP beads (Beckman Coulter, Brea, California, USA), and beads were washed twice with 200 µL 75% EtOH. Final PCR on the pooled library was conducted in eight separate reactions with 5x NEB Master Mix as follows: 95°C for 5 min; 18 cycles of 98°C for 30 s, 65°C for 30 s, 72°C for 17 s; 72°C for 5 min. Final library cleanup was performed with 44 µL AMPure XP beads, which were washed twice with 200 µL 75% EtOH. Each library was eluted in 40 µL of 10 mM Tris‐HCl pH 8.0 and quantified on a Qubit fluorometer (Life Technologies, Carlsbad, California, USA), before pooling the four highest concentration reactions for sequencing. The final libraries were sequenced by Novogene (Sacramento, California, USA) on two lanes of an Illumina HiSeq. 4000 (Illumina, San Diego, California, USA) for 150 bp paired‐end reads.

Raw sequence reads were processed to generate a filtered SNP data set for population structure analyses. Fast‐GBS version 2 was implemented for discovery of genome‐wide variants (Torkamaneh et al., 2020). Within the pipeline, Sabre (https://github.com/najoshi/sabre) was used to sort and filter barcodes, and Cutadapt (Martin, 2011) was used to trim reads of the barcode region. Paired‐end reads were then aligned to the *C. sativa* V2 reference genome (Kagale et al., 2014) using BWA. Four different SNP data sets were generated: all samples in the data set; only samples clustering with *C. sativa* and 2n = 40 *C. microcarpa*; only 2n = 38 *C. microcarpa*; and only *C. rumelica*. Variants were searched with Platypus variant caller (https://github.com/andyrimmer/Platypus) and PLINK (Purcell et al., 2007) was then used to generate a VCF file in which an initial genotyping filter of 0.2 was employed to filter any variants with >20% missing data. Next PLINK was run to filter variants accordingly: Minor allele frequency (‐‐maf) = 0.05, Genotyping (‐‐geno) = 0.1, Missing data per individual (‐‐mind) = 0.5. Filtering for the data set in which all individuals were included used a Missing data per individual = 0.6 to accommodate more samples passing filtering. Two samples were removed due to excess missing data; 39,473 variants were removed due to missing genotype data; 2951 variants were removed due to deviations in the Hardy‐Weinberg exact test; and 3648 variants were removed based on the minor allele threshold.

For a separate analysis, the data set was further trimmed to include only the cluster of samples including 2n = 40 *C. microcarpa* and *C. sativa*. Filtering was the same as above, except that a more stringent Missing data per individual = 0.5 was applied. One individual was removed due to excess missing data; 196,444 variants were removed due to missing genotype data; 10,067 variants were removed due to deviations in the Hardy‐Weinberg exact test; and 63,762 variants were removed based on the minor allele threshold. The final data set included 10,737 variants and 110 individuals (50 *C. sativa* and 60 *C. microcarpa*). Additional analyses conducted for the groups including only 2n = 38 *C. microcarpa* and *C. rumelica* were performed using the same parameters as the *C. sativa*/*C. microcarpa* analysis, and filtering results are reported in Appendix S2.

### Population genetic analyses

Population genetic structure was determined using ADMIXTURE version 1.3.0 (Alexander et al., 2009) on the final data sets of 2n = 40 *C. microcarpa* and *C. sativa* only (Appendix S3) and 2n = 38 *C. microcarpa* only (Appendix S4) with *K* values from 1 to 10. Cross validation error scores were obtained from ADMIXTURE for each *K* value to assess the optimal *K* values (Appendix S5). A neighbor‐joining tree was generated in TASSEL version 5.2.72 (Bradbury et al., 2007) using the final SNP data set for the 2n = 40 genetic cluster. Measures of population genetic diversity and differentiation were obtained using Stacks version 2.53 (Rochette et al., 2019). Reads aligned to the *C. sativa* reference genome (.bam files) generated in Fast‐GBS version 2 were used in the Stacks program gstacks to identify and phase SNPs into a set of haplotypes. The catalog of loci generated by gstacks was then used in the populations program within Stacks. To calculate population‐level diversity statistics, all reference mapped reads were used to approximate genome‐wide nucleotide diversity (π). *F*_ST_ statistics were calculated in accordance to Weir and Cockerham's *F*_ST_ on the final filtered SNP data sets using VCFtools version 0.1.5 (Danecek et al., 2011). When grouping individuals into populations for pairwise *F*_ST_ analyses, individuals with <70% of ancestry from a single population were labeled as admixed (Table 1).

**Table 1:** Pairwise mean *F*_ST_ between *Camelina sativa* and *C. microcarpa* (2n = 40) genetic populations. Individuals were assigned to a genetic population if they were >70% assigned to that population based on ADMIXTURE. If an individual's population structure identity was <70% for a single genetic population it was labeled admixed.

	*C. sativa*	*C. microcarpa* Armenia	*C. microcarpa* Georgia
***C. microcarpa*** **Armenia**	0.1166		
***C. microcarpa*** **Georgia**	0.1615	0.0731	
**Admixed**	0.0968	0.0171	0.0373

### Analysis and mapping of archaeological sites

Published archaeological literature was surveyed to provide insights on the origin and spread of cultivated and weedy *C. sativa* (see references in Appendix S6). Studies that reported reliable identifications and included additional information such as time period were included for mapping. When available, information such as likely species (i.e., *C. microcarpa* or *C. sativa*), numbers of seeds or remains found, and whether the report suggests a weedy or cultivated context were also included (Appendix S7). Time periods were grouped by millennia (8th millennium BCE to 2nd millennium CE) and sites were shaded accordingly on a map to visualize camelina usage over time across its range. The locations of overlapping sites were shifted slightly to better visualize neighboring sites. The data were mapped in ArcMap version 10.6 (ESRI, Redlands, California, USA).

## RESULTS

### Analysis of all sampled *Camelina*

**Figure 2:**
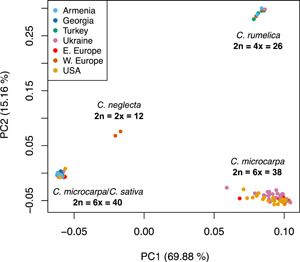
PCA generated from the final SNP data set for 183 *Camelina* individuals that passed filtering. Colored dots represent individual's country of origin and ellipses represent taxonomic groups, as indicated including observed karyotypes within those groups.

After data filtering, genotyping‐by‐sequencing yielded a data set of 2936 variants across 183 accessions (Appendix S2). Principal component analysis (PCA) using all genotyped samples indicated a clustering of samples by taxonomic and cytotypic groups, with four distinct clusters present: all 2n = 40 *C. microcarpa* and *C. sativa* individuals; *C. neglecta*; 2n = 38 *C. microcarpa*; and *C. rumelica* (Figure 2). This pattern of differentiation mirrors a previous GBS sequencing project that also included these domesticated and wild taxa (Chaudhary et al., 2020). The two diploid *C. neglecta* samples were clustered between the three polyploid clusters; this is also an expected result, considering that all other clusters studied here represent polyploids of hybrid origin involving a parental *C. neglecta* ancestor (Mandáková et al., 2019; Brock et al., 2022).

Among the *C. microcarpa* samples, those that grouped into the 2n = 40 cluster with the crop species included all 51 of 51 South Caucasus (including eastern Turkey) individuals, 6 of 31 individuals from the United States, and 3 of 31 individuals from Ukraine; none of the 8 *C. microcarpa* individuals from the rest of Europe grouped into the 2n = 40 cluster. The 2n = 38 *C. microcarpa* cluster included the remaining samples from the United States, Ukraine, and Europe. Notably, the 2n = 38 *C. microcarpa* is separated from the 2n = 40 *C. microcarpa* and *C. sativa* cluster along PC1 (accounting for nearly 70% of the variance) and is roughly as dissimilar in PCA space as *C. rumelica* is to the 2n = 40 group (Figure 2). This observation further supports the 2n = 38 cytotype of *C. microcarpa* as being highly genetically distinct from the 2n = 40 cytotype.

### Analysis of 2n = 40 *C. microcarpa* and *C. sativa* strongly supports a Caucasus domestication origin

**Figure 3:**
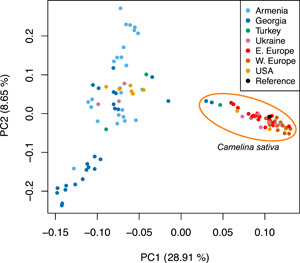
PCA generated from the final data set of 10,737 SNPs from 110 individuals, including only 2n = 40 *Camelina microcarpa* and *C. sativa*. Colored dots represent individual's country of origin.

Samples belonging to *C. sativa* and the closely related 2n = 40 *C. microcarpa* individuals were included in a separate analysis to assess population structure and differentiation for the purposes of identifying a geographical origin of *C. sativa* domestication. The final SNP data set included 110 individuals and 10,737 SNPs. A PCA generated by PLINK shows *C. sativa* accessions as tightly clustered together and separated from the more loosely arrayed *C. microcarpa* along PC1 (representing 28.91% of variance) (Figure 3). Three *C. sativa* samples collected from outside agricultural contexts in Georgia and eastern Turkey (Ames 31231, Ames 31232, and JRB 153) are clustered with the rest of *C. sativa* but with closer proximity to *C. microcarpa* along PC1, suggesting that these could be protodomesticates or the result of crop‐weed hybridization. Two subsets of *C. microcarpa* accessions predominantly originating from Georgia and Armenia are clustered separately from the remainder of *C. microcarpa* accessions along PC2 (explaining 8.65% of variance). Interestingly, despite occupying relatively close geographic proximities in the Caucasus, subgroups of C*. microcarpa* from Georgia and Armenia are separated along PC2, indicating genetic differentiation between these groups.

**Figure 4:**
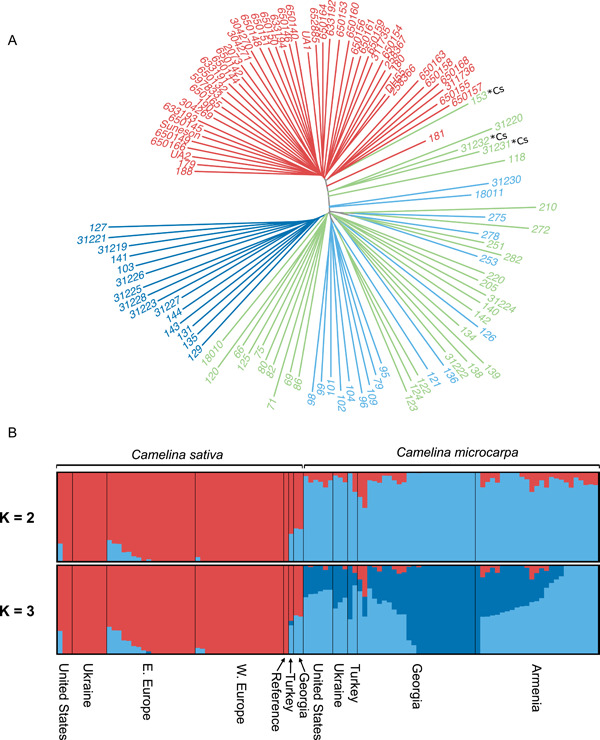
Analysis of relatedness and population structure of 2n = 40 *Camelina microcarpa* and *C. sativa* individuals. (A) Neighbor‐joining tree with individuals colored according to population genetic identity >70%; *C. sativa*, red; *C. microcarpa* Armenia, light blue; *C. microcarpa* Georgia, dark blue; hybrid (<70% population identity to any genetic group), green. Admixed *C. sativa* individuals denoted with ‘*Cs’. (B) Admixture plots at *K* = 2 and *K* = 3 with morphologically determined *C. sativa* (left) and *C. microcarpa* (right) split.

Population structure of the 2n = 40 accessions inferred by ADMIXTURE revealed *K* = 3 as the best fit indicated by the lowest CV error (Appendix S5). The three genetic populations correspond to the crop *C. sativa*, a primarily Georgian population of *C. microcarpa*, and a primarily Armenian population of *C. microcarpa* (Figure 4). Patterns of genetic structure among 2n = 40 accessions are broadly consistent with the PCA results, such that an entirely *C. sativa* subpopulation and two subpopulations of *C. microcarpa* from Armenia and Georgia are observed, matching PCA clustering patterns described above. Results from ADMIXTURE show low amounts of admixture in most *C. sativa*, and almost exclusively with the Armenian genetic population of *C. microcarpa* at *K* = 3. The three *C. sativa* confirmed to be collected outside of agricultural contexts all show comparatively high levels of admixture, whereas Western European and Ukrainian individuals show almost none. Among *C. microcarpa* accessions, all represented geographic regions show at least one individual to have at least some admixture with *C. sativa*.

The *F*_ST_ estimate between *C. microcarpa* and *C. sativa* was 0.09416, suggesting modest genetic differentiation between the crop and its pre‐domesticate. Genetic diversity within *C. microcarpa* (π = 0.00021) was found to be higher than *C. sativa* (π = 0.00013), consistent with a domestication bottleneck. Pairwise *F*_ST_ between genetic populations as defined by ADMIXTURE results (subgrouped into *C. sativa*, Armenian *C. microcarpa*, and Georgian *C. microcarpa*) show that admixed individuals (<70% identity with a single genetic population) have the lowest genetic differentiation (*F*_ST_ = 0.09680) with *C. sativa*, followed by the Armenian *C. microcarpa* population (*F*_ST_ = 0.11664) and the Georgian *C. microcarpa* population (*F*_ST_ = 0.16154) (Table 1). Taken together, these findings are consistent with an Armenian origin of modern *C. sativa* samples.

### Analysis of 2n = 38 *Camelina macrocarpa*

Of the total 120 *C. microcarpa* samples sequenced, 61 were grouped into the 2n = 38 cluster in the PCA (Figure 2). Cytotype determinations have previously been performed on 18 of these samples, all of which were confirmed to be 2n = 38 (Brock et al., 2022). To further assess population structure within this group, the SNP pipeline was run separately to generate a data set of 60 individuals and 24,847 SNPs (Appendix S2). The lowest cross‐validation error was observed at *K* = 1 (0.42459); however, it was only negligibly higher at *K* = 2 (CV error = 0.42926), and we consider *K* = 2 to make the most biological sense, given the pattern of geographic clustering that emerges at this *K* value (Appendices S4, S5). ADMIXTURE results show clear separation within the 2n = 38 group at *K* = 2, such that one genetic population includes individuals from southern Ukraine and the remaining population is predominantly represented by north Ukrainian, European, and U.S. samples (Appendix S8). This result is consistent with a geographical trend previously observed in a subset of these samples (Brock et al., 2020). These results indicate that the 2n = 38 cytotype of *C. microcarpa* predominates in Europe and the United States (Appendix S9) and, of the areas sampled, appears to be most diverse in Ukraine.

### Archaeological literature suggests a Southwest Asian domestication origin for camelina

**Figure 5:**
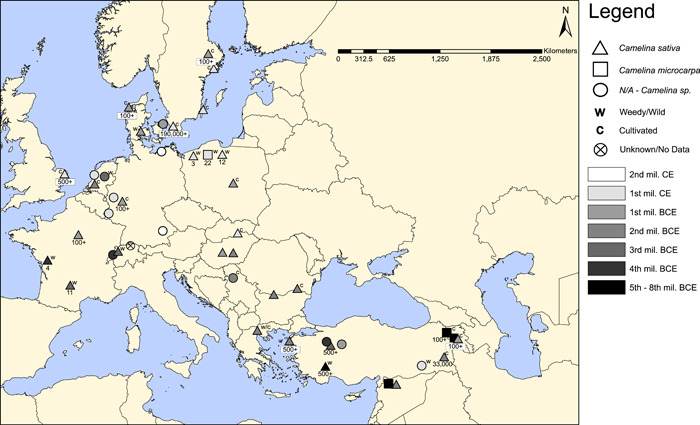
Map of archaeological records for *Camelina* across Europe and Western Asia. Points are mapped according to the approximate location of archaeological sites. Triangles = *C. sativa*; squares = *C. microcarpa*; circles = *Camelina* sp. or unidentified. Letters above and to the left or right of sites indicate that the plants are likely cultivated (C) or weedy/wild (W). Numbers below sites represent approximate number of *Camelina* seeds recovered at sites for which this information was available. Sites are shaded to denote approximate age of the site or sample, from black (oldest) to white (youngest).

We searched the literature for available archaeological sources for *Camelina* and produced a map representing the approximate locations, ages, number of seed remains found, cultivation status, and taxonomic identity when available (Figure 5). Given the high oil content of *Camelina* seeds, it may be assumed that these remains would be less likely to char and become preserved in the archaeological record when compared to grain crops (Wilson, 1984; Märkle and Rösch, 2008; Toulemonde et al., 2010). The earliest archaeological evidence of *C. microcarpa* comes from the 8th millennium BCE in Syria and 6th millennium BCE in Armenia (Hovsepyan and Willcox, 2008), with the latter providing evidence of intentional cultivation or potential foraging for this wild species. For *C. sativa*, the earliest record is from Turkey at Kuruçay Höyük around 4000 BCE (Stroud, 2016). These findings show that the earliest documentation of collection/cultivation of *C. microcarpa* and earliest records of *C. sativa* occur in Southwest Asia before spreading to Europe and finally Scandinavia.

In contrast to the South Caucasus region, the earliest *C. sativa* seeds recovered from Europe were from 3650–3350 BCE in western France. Notably, these early records are characterized by few camelina seeds, which are always in association with flax and wheat, indicating a high likelihood that they represent weedy seed contaminants (Bouby, 1998). Clear evidence of *C. sativa* cultivation in Europe first appears around 1600–1000 BCE, in the Late Bronze Age to early Iron Age (Appendix S7; Figure 5). Together, these findings suggest that *C. sativa* was often found as a weed in Europe at least two millennia prior to evidence of intentional cultivation and its subsequently widespread use in Iron Age Europe as an oilseed crop.

## DISCUSSION

Following 20th‐century declines in camelina cultivation and the large‐scale abandonment of cultivars, renewed interest in this oilseed crop for biofuels and other applications has spurred ongoing efforts to better understand its genomic composition and evolutionary origins (Kagale et al., 2014; Brock et al., 2018, 2022; Mandáková et al., 2019; Žerdoner Čalasan et al., 2019; Chaudhary et al., 2020). While this work has clarified phylogenetic relationships among *Camelina* species and characterized subgenome origins of the allohexaploid crop lineage, important questions have remained unanswered regarding camelina's geographical domestication origins and early cultivation history. Drawing from both genetic and archaeological data, the present study provides several key insights into this domestication history. We find evidence of genetic similarity between a subpopulation of Armenian *C. microcarpa* and *C. sativa*, supporting a South Caucasus origin of the crop. Additionally, through an analysis of archaeological data, we find support for use of the pre‐domesticate in Armenia preceding domestication and the subsequent spread of *C. sativa* throughout Europe. We discuss the implications of these findings below.

### Genetic evidence for a South Caucasus origin of domesticated camelina

The preponderance of data, including next‐generation sequencing and cytogenetic data, shows that *C. sativa* (2n = 40) was domesticated from the 2n = 40 cytotype of *C. microcarpa* (Brock et al., 2018), and that the genomes remain structurally conserved (Mandáková et al., 2019; Chaudhary et al., 2020). Thus, the rarity of 2n = 40 *C. microcarpa* in Europe indicates that this region is unlikely to have been the origin of *C. sativa* domestication.

Genetic clustering of wild‐collected *C. sativa* from Turkey and Georgia in PCA space is closest to the wild progenitor (Figure 3), although only these three definitively wild‐collected individuals of *C. sativa* were available for this study, due to its rarity in weedy and feral contexts. These individuals also show evidence of shared genetic composition with *C. microcarpa* (Figure 4). All other *C. sativa* individuals show only low levels of admixture, and almost exclusively from the Armenian *C. microcarpa* subpopulation (Figure 4). On the other hand, many *C. microcarpa* individuals show low levels of admixture from *C. sativa* (Figure 4), although it is not clear if the observed genetic admixture is a result of introgression or shared ancestry. Notably, at *K* = 4 (though with a higher CV error; Appendix S5) a second genetic population of *C. sativa* is evident such that individuals generally cluster into Eastern and Western European subpopulations (Appendix S3), similar to observations reported in a recently published genome resequencing study in the crop (Li et al., 2021) and a biogeographic ETS sequencing study in the genus (Žerdoner Čalasan et al., 2019).

Genetic differentiation between the crop and geographically diverse populations of its wild progenitor species provides a signal of potential Armenian origin. Pairwise genetic differentiation between *C. sativa* and the Armenian *C. microcarpa* population (*F*_ST_ = 0.11664) was lower when compared to the Georgian *C. microcarpa* population (*F*_ST_ = 0.16154). Additionally, individuals belonging to the Armenian genetic population group more closely to *C. sativa* in a phylogenetic context, compared to the Georgian individuals (Figure 4). Those individuals of the closest phylogenetic placement to *C. sativa* are from Armenia (118), Georgia (31220, 31230), and Turkey (18011). These results contradict a previous genetic study concluding a likely Ukrainian origin (Ghamkhar et al., 2010), and are further supported by our observation that the 2n = 40 cytotype of *C. microcarpa* is rarely found in Ukraine. Broader geographic sampling may result in additional clarity and further population genetic structuring within *C. microcarpa*, though the current evidence indicates a South Caucasus origin, specifically in the region of present‐day Armenia. We acknowledge that the present study is biased with respect to geographic sampling. To provide additional resolution, future studies should attempt to incorporate diversity of *C. microcarpa* and *C. sativa* from Central and Western Europe, Syria, Iraq, Iran, and the eastern Eurasian steppe.

### Genetic clustering of *Camelina microcarpa* cytotypes and their geographic distributions

Previous studies have shown the prevalence of the 2n = 38 cytotype of *C. microcarpa*, which is highly genetically distinct (Brock et al., 2020; Chaudhary et al., 2020) and has a unique polyploidization history from the 2n = 40 cytotype (Chaudhary et al., 2020; Brock et al., 2022). The current study finds that *C. microcarpa* samples originally obtained from Europe and deposited in the USDA GRIN collection always group with 2n = 38 *C. microcarpa* (Figure 2). Among all 2n = 38 *C. microcarpa* individuals, we find evidence of a distinct south Ukrainian genetic population (Appendix S8), consistent with our previous findings (Brock et al., 2020). Only rarely is the 2n = 40 *C. microcarpa* cytotype found in Ukraine and the United States, and it is probable that this cytotype is present in Europe at low frequencies. In contrast, 2n = 40 *C. microcarpa* is common in the Caucasus where the 2n = 38 cytotype is absent (Appendix S9).

### Archaeological evidence supporting a South Caucasus origin of *C. sativa*

Visualizing the archaeological finds of *Camelina*, two points become clear: (1) The earliest documented intentional collection/cultivation of *C. microcarpa* occurred in Armenia, with the earliest evidence of *C. sativa* in Southwest Asia before spreading to Europe and finally Scandinavia. And (2) *C. sativa* was often found as a weed in Europe, likely due to its association with flax, long before evidence of intentional cultivation and its subsequently widespread use in Iron Age Europe.

Understanding the distribution and use of cultivated and weedy *Camelina* is made difficult by the fact that oilseeds generally carbonize less well than other types of seeds such as grains, and are less well preserved in the archaeological record (Wilson, 1984; Märkle and Rösch, 2008). Moreover, we speculate that any camelina intentionally cultivated for its oil likely would have been processed shortly after harvest, further reducing the likelihood of the charring of intact seeds. Thus, it is reasonable to conclude that use of camelina in ancient times may have been more prevalent than the archaeological record suggests.

The earliest known archaeological records of *Camelina* anywhere in the world are of *C. microcarpa* in the 8th millennium BCE at Djade in Syria (Rivera et al., 2011) and in the 6th millennium BCE Armenian sites of Aratashen and Aknashen (Hovsepyan and Willcox, 2008). While these earliest records comprise the wild *C. microcarpa*, the two latter examples show the presence of separated valve impressions indicating that they were likely not weedy but collected or cultivated intentionally and processed for the oil‐rich seeds in Armenia (Hovsepyan and Willcox, 2008). Additionally, the frequency of *C. microcarpa* samples at these sites indicates that they were of economic importance. Very close to these early sites, at the Urartian site of Teishebaini (Aka Karmir Blur) large quantities of *C. sativa* seeds were found dating to 700–600 BCE (Tumanyan, 1944). However, even before this, large numbers of *C. sativa* seeds were recovered in a pot in Syria at Hadidi around 2300–1200 BCE (van Zeist and Bakker‐Heeres, 1985) and from Turkey at Kuruçay Höyük around 4000 BCE (Stroud, 2016), Küllüoba around 3000–1200 BCE (Çizer, 2015), Kumtepe and Troy around 2100–1700 BCE (Riehl, 1999), and Yocantepe around 1000 BCE (Dönmez and Belli, 2007). Thus, it appears that the earliest stages of domestication had begun around or before the 6th millennium BCE, and that *C. sativa* had been domesticated in Southwest Asia by or before the 4th millennium BCE. How *C. sativa* arrived in Europe may be difficult to ascertain. It has previously been suggested that flax weeds, including *C. sativa*, spread into Europe with the movement of flax (Nesbitt, 1996). Finally, because the earliest archaeological record of *C. microcarpa* originates from Syria, it will be valuable to survey genetic variation in this region and determine if it might, in fact, be the center of domestication.

### Early studies and historical uses of camelina

Various forms of *C. sativa* were described in the early 20th century, yet these are seldom discussed or examined in modern work. It is likely that these forms arose through various selective regimes in the agricultural context (e.g., as a flax weed or weed of other crops, through co‐cultivation with flax, or through cultivation as a standalone crop). Several detailed descriptions of morphotypes of *C. sativa* come from a handful of early 20th‐century authors, who described numerous forms of the plant in agricultural and weedy contexts. One such form, designated ‘*C. caucasica*,’ was a smaller‐seeded variety that had been reported to be frequently grown in Armenia in co‐culture with oilseed flax (linseed) and at higher elevations in monoculture (Sinskaya and Beztuzheva, 1931). Another form, adapted as a weed and crop mimic to dense fiber flax sowings (‘*C. linicola*’), was reported to be the tallest, least branched, and largest‐fruited form; this form was never adopted as a standalone crop because it was found to be unsuitable for cultivation (Sinskaya and Beztuzheva, 1931). Another variety, ‘*C. glabrata*,’ was described as a weed of oilseed flax, as well as of other crops such as oats and barley (Sinskaya and Beztuzheva, 1931). In the case of rare types of “shattering flax” or ‘*Linum crepitans*,’ a similar type of shattering camelina (‘*C. crepitans*’) was described, which dehisced fruit valves in early stages of maturation. By the 1960s, it appears that many of these weedy varieties had become rare, or altogether extinct, including ‘*C. crepitans*’ and ‘*C. linocola*’ (Sinskaya, 1969). Sinskaya also notes that winter varieties had become less common by this time, although they could still occasionally be found as weeds of other winter crops. Within the Former Soviet Union, much attention was paid to these weedy forms infesting flax and other agricultural crops, but it is clear that *C. sativa* was also cultivated regularly as an oilseed crop, and, at least in some areas, this was frequently done in intentional co‐culture with oilseed flax (Sinskaya and Beztuzheva, 1931).

Looking back at these early observations, it is difficult to assess the relative roles of phenotypic plasticity vs. genetic architecture in producing this varietal diversity. Notably, however, Tedin and Zinger had experimentally demonstrated that at least some genetic components underlie the morphological differences in these various forms of *C. sativa* (Zinger, 1909; Tedin, 1925), indicating that this variation was not solely plasticity in response to the growing environment. In our opinion, none of the variation within cultivated camelina that once existed or that exists today would be sufficient to meet any species concept; nevertheless, variation observed in contemporary *C. sativa* represents a fraction of the diversity discussed in those original reports. Anecdotally, variation in leaf shape and margins, fruit size, density of fruits, and inflorescence branching patterns observed in historical herbarium specimens from the 19th and early 20th centuries is rarely represented in modern cultivars (J. Brock, personal observation). This observation is consistent with previous descriptions by early Soviet scientists who documented a wide range of morphotypes of *C. sativa* in the early 20th century. One explanation for the apparent rapid disappearance of diverse *C. sativa* phenotypes is a high oil content in the seeds, which oxidizes over time, leading to short‐term viability of the seeds and the propensity for cultivar loss without continuous propagation.

The origins of *C. sativa* were poorly understood in the early research on camelina. Vavilov had suggested a Mediterranean origin of cultivated forms of many crop plants, including flax (Vavilov, 1926). Building on Vavilov's findings and based partially on observations that Western Asia is dominated by small‐seeded camelina (i.e., *C. microcarpa*), Sinskaya considered the Mediterranean region as the birthplace of *C. sativa* (Sinskaya, 1928). At least one other author has suggested an Eastern Mediterranean origin of *C. sativa* (Rivera et al., 2011). However, Sinskaya also suggested that at least one cultivated form of camelina in Armenia arose from local populations of *C. microcarpa*, supported by the discovery of a small vessel full of *C. sativa* seeds found at Teishebaini, an ancient Urartian city in Armenia (Tumanyan, 1944; Sinskaya, 1969).

Camelina had numerous uses, including its occasional use in England as a forage for sheep, and the use of camelina stems in France for making brooms and roof thatching, among other potential uses involving its low‐quality fiber (Sinskaya, 1928). In Armenia, camelina oil was both consumed and used for lighting, while the fibrous stems were used for spinning (Gabrielian and Zohary, 2004; Rivera et al., 2011). Remnant fruit valves from the threshing process for camelina were also used to temper pottery, and may have had other technical uses, possibly even in the tanning of leather (Bakels et al., 2019). In Europe and Scandinavia, camelina was consumed in porridge and added to bread in a similar manner to modern sesame seeds (Hatt, 1937). In fact, seed remains of *C. sativa* were discovered in the stomachs of the well‐preserved bog bodies of Tollund Man and Huldremose Woman from Iron Age Denmark, showing consumption of the oilseed in porridge or gruel (Helbaek, 1950; Holden, 1997). The residual seed meal of camelina processed for oil was used predominantly as a protein and oil‐rich fodder for a variety of livestock (Wacker, 1934; Andersson and Olsson, 1950). In Ukraine, traditional uses include using bundled stems as brooms, extracting seed oil for use in food and on salads, and use of the seed‐meal for livestock feed (J. Brock, personal observation).

Cultivation of *C. sativa* may not have slowed or ended evenly throughout Europe. Between 1861 and 1882, it was noted that the area of camelina cultivation had decreased by two‐thirds in France and Germany (Maurizio, 1932; Bouby, 1998), long before the supplanting of camelina with rapeseed and other oilseed crops in European agriculture following World War II. By 1900 the crop had almost entirely disappeared in France (Martin, 1947), though it was still reportedly grown to some extent from this time through to the 1930s in Holland, Belgium, the Balkans, and Russia from the Caucasus to Siberia (Wacker, 1934). In the decade immediately after the end of World War II, camelina was still cultivated, likely to a lesser degree, in some parts of Europe including Poland and Sweden (Zubr, 1997). At least some areas may have never completely ceased *C. sativa* cultivation, as evidenced by continued (albeit extremely rare) small‐scale rural cultivation in Ukraine (J. Brock, personal observation) and Slovenia (Rode, 2002). Only since the late 20th century has camelina cultivation resumed, albeit sporadically, in North America and Europe. Many sources suggest a dramatic loss and even putative extinction of *C. sativa* growing in weedy or feral contexts in North America (McGregor, 1985; Al‐Shehbaz and Beilstein, 2010) and Europe (Mirek, 1997; Hulina, 2005; Heller, 2010). However, although rare, *C. sativa* may still be found in North America (Martin et al., 2017), Europe (www.inaturalist.org), the Caucasus (USDA GRIN), and the Eurasian steppe (Žerdoner Čalasan et al., 2019). These contemporary records may be indicative of feral escapes or even long‐standing feral and/or weedy populations. The collection of *C. sativa* that has remained in continual cultivation or is confirmed to be a weed of flax or other crops would provide the basis for future studies examining the genetic architecture of the various weedy and crop‐like traits originally described by Sinskaya, Tedin, and Zinger and could prove a valuable source of genetic diversity for modern breeding.

### Secondary crop vs. primary crop origin of *C. sativa*

Together with the ethnobotanical and archaeobotanical review provided above, the notion of *C. sativa* being co‐opted as a crop only after its evolution as a weedy specialist of flax fields is put into question. Instead, intentional cultivation of *C. sativa*, and even its pre‐domesticate *C. microcarpa*, appears likely. Firstly, evidence for intentional collection or cultivation of *C. microcarpa* at two archaeological sites in 6th millennium BCE Armenia suggests that the pre‐domesticate was valuable in its own right. Our previous study showed that although *C. sativa* has significantly increased seed oil content relative to *C. microcarpa*, their seed oil compositions are largely indistinguishable (Brock et al., 2020). Thus, the relative nutritive content and chemical properties (for use as fuel) are little changed, supporting the idea that *C. microcarpa* would have represented a valuable forage. We are currently limited in our capacity to provide inferences on whether the target of selection during domestication was on weedy flax mimics, or instead on *C. microcarpa* and *C. sativa* that was being sought out as an oil source even before the species had become adapted as a flax mimic, as these changes (especially increased seed size and reduced shattering) would have arisen in either case.

Some cultures may have switched between cultivation of flax and camelina at different times (Karg, 2012), perhaps in response to changing environmental conditions or biotic stressors exhibited on one or the other through time. There even exists evidence of flax and camelina being stored and processed separately at the same site (Larsson, 2013). However, the fact that *C. sativa* and flax were, at least sometimes, grown in intentional co‐culture suggests a potentially novel explanation that there was some benefit to mixed cropping. Previous descriptions of *C. sativa* seeds in archaeological records often record it as a weed, yet it sometimes appears in a 1:1 ratio with flax, suggesting intentional co‐culture (Riehl, 1999). One reason for this could have been an increased hardiness or disease resistance in camelina compared to flax that served as a bet‐hedge. The often‐overlooked aspect of intentional flax and camelina co‐culture deserves further investigation.

In summary, our evidence suggests that *C. sativa* originated from *C. microcarpa* from the South Caucasus, possibly Armenia, concordant with archaeological data and historical descriptions of intentional cultivation of *C. sativa* in Armenia. However, additional sampling from Syria, Iraq, and Iran may provide evidence that, in fact, domestication occurred farther south than our data suggest. Evidence of camelina's consumption before and after domestication and its use in intentional co‐culture with flax suggests that the well‐circulated notion that *C. sativa* was put into cultivation only after having evolved as a weed of flax may not be adequately supported.

### Implications for future improvement of camelina crops

The discovery of a widespread cytotype of *C. microcarpa* (2n = 38), a cryptic taxon with high morphological similarity to the 2n = 40 cytotype, has many implications for modern camelina cultivation. The variant cytotype is unlikely to form viable hybrid offspring with 2n = 40 *C. microcarpa* or *C. sativa* due to their dissimilar cytotypes. An early crossing experiment in Sweden (Tedin, 1925), and a more recent experiment in France (Tepfer et al., 2020), noted that *C. microcarpa* and *C. sativa* are largely infertile, an observation that would be expected if the *C. microcarpa* studied was of the 2n = 38 cytotype. Additional studies should be conducted with a specific focus on the viability of offspring generated from *C. sativa* and the 2n = 38 cytotype of *C. microcarpa*. If these are found to be sexually incompatible, it would potentially facilitate the deployment of transgenic *C. sativa* in areas where the genetically compatible 2n = 40 cytotype is rare or absent. The threat of transgene escape may be lower in the United States and Europe than previously thought, considering that most sampled populations there are of the 2n = 38 cytotype. Further, Europe is unlikely to be a valuable source of genetic variation for breeding, given that any future 2n = 40 *C. microcarpa* found there would likely be of more recent introduction and harbor less genetic variation than those in Southwest Asia. Finally, our results show that genetically differentiated subpopulations of 2n = 40 *C. microcarpa* exist, and their diversity may prove valuable for future breeding and crop improvement endeavors. The implications of this work are important and timely, as ever‐increasing interest in redevelopment of *C. sativa* as a sustainable oilseed crop is taking hold in Europe (Zanetti et al., 2021) and the United States (Eynck and Falk, 2013; Obour et al., 2015).

## CONCLUSIONS

We provide the first genetic evidence of genetic relatedness between populations of *C. microcarpa* and *C. sativa*, suggesting a South Caucasus, possibly Armenian, origin of domestication. Archaeological records of *Camelina* in Europe and Southwest Asia support Armenia as an early site of cultivation for the pre‐domesticate with the earliest findings of *C. sativa* in Southwest Asia. The distribution of two *C. microcarpa* cytotypes confirms that the pre‐domesticate (2n = 40 cytotype) is widely present in the Caucasus but infrequently found in Europe. Finally, we encourage additional investigation into the potential nature of camelina and flax co‐culture and the notion that camelina is a secondary crop.

## AUTHOR CONTRIBUTIONS

J.R.B. performed molecular work, analyzed data, and generated maps and figures. M.M.R. curated an overview of archaeological records and revised the final manuscript. K.M.O. and J.R.B. conceptualized the study and wrote the manuscript.

## Supporting information


**Appendix S1**. Sample information for individuals sequenced in this study, including accession information, barcodes used in sequencing, collection information, confirmed cytotype (where available), and GenBank accession number.

**Appendix S2**. Filtering results for all SNP data sets, including the total number of individuals, number of individuals passing filtering, variants remaining and removed after various filtering steps, and final number of variants included in each data set.

**Appendix S3**. ADMIXTURE results of the final 2n = 40 *Camelina microcarpa* and *C. sativa* data set run at *K* = 1–10 and displayed with pong. Individuals are grouped based on morphological identity with *C. sativa* on the left and *C. microcarpa* on the right, with individuals subgrouped by country of origin.

**Appendix S4**. ADMIXTURE results of the final 2n = 38 *Camelina microcarpa* data set run at *K* = 1–10 and displayed with pong. Individuals are grouped based on country of origin.

**Appendix S5**. Cross‐validation error results from ADMIXTURE runs from *K* = 1–10 for (A) *Camelina sativa* and 2n = 40 *C. microcarpa* and (B) 2n = 38 *C. microcarpa* data sets.

**Appendix S6**. Supplementary references for archaeological literature survey. References provided here correspond to those cited in Appendix S7 and comprise the body of literature used to determine the geographic scope of *Camelina* archaeological findings and their timing and cultivation status.

**Appendix S7**. Summary of published archaeological literature including reports of *Camelina* spp. Where available information about the archaeological site was recorded, including region, site name, time period, number of seeds or remains found, species, and cultivation status.

**Appendix S8**. Analysis of final SNP data set for 60 individuals clustering with the 2n = 38 *C. microcarpa* group. (A) PCA, colored dots represent individual's country of origin. (B) Admixture plot at *K* = 2; USA and Europe genetic group, pink; S. Ukraine genetic group, brown.

**Appendix S9**. Mapped locations of 2n = 38 *C. microcarpa*, pink circles with black centroid and 2n = 40 *C. microcarpa*, blue circles, from (A) United States and (B) Europe and the Caucasus. USDA GRIN accessions from Europe were approximately mapped due to missing coordinate data.

## Data Availability

The data sets used in this article are available in the GenBank repository and can be accessed at the Sequence Read Archive (SRA) at https://www.ncbi.nlm.nih.gov/sra with accessions numbers SRR12391781–SRR12391863 and SRR18493181–SRR18493286 and under BioProject IDs PRJNA655452 and PRJNA820288.
