# Supplementary material for: Molecular and archaeological evidence on the geographical origin of domestication for Camelina sativa
Source: Am J Bot. 2022 Jul 11;109(7):1177–90. doi: 10.1002/ajb2.16027 (PMC9542853; doi:10.1002/ajb2.16027)
Supplement: Supplementary file 3 — Appendix S3. ADMIXTURE results of the final 2n = 40 Camelina microcarpa and C. sativa data set run at K = 1–10 and displayed with pong. Individuals are grouped based on morphological identity with C. sativa on the left and C. microcarpa on the right, with individuals subgrouped by country of origin. [file AJB2-109-1177-s008.docx]

**Appendix S3**: ADMIXTURE results of the final 2n = 40 *C. microcarpa* and *C. sativa* dataset run at K = 1 – 10 and displayed with pong. Individuals are grouped based on morphological identity with *C. sativa* on the left and *C. microcarpa* on the right, with individuals sub-grouped by country of origin.
