# Supplementary material for: Molecular and archaeological evidence on the geographical origin of domestication for Camelina sativa
Source: Am J Bot. 2022 Jul 11;109(7):1177–90. doi: 10.1002/ajb2.16027 (PMC9542853; doi:10.1002/ajb2.16027)
Supplement: Supplementary file 4 — Appendix S4. ADMIXTURE results of the final 2n = 38 Camelina microcarpa data set run at K = 1–10 and displayed with pong. Individuals are grouped based on country of origin. [file AJB2-109-1177-s004.docx]

**Appendix S4**: ADMIXTURE results of the final 2n = 38 *C. microcarpa* dataset run at K = 1 – 10 and displayed with pong. Individuals are grouped based on country of origin.
