# Supplementary material for: Molecular and archaeological evidence on the geographical origin of domestication for Camelina sativa
Source: Am J Bot. 2022 Jul 11;109(7):1177–90. doi: 10.1002/ajb2.16027 (PMC9542853; doi:10.1002/ajb2.16027)
Supplement: Supplementary file 5 — Appendix S5. Cross‐validation error results from ADMIXTURE runs from K = 1–10 for (A) Camelina sativa and 2n = 40 C. microcarpa and (B) 2n = 38 C. microcarpa data sets. [file AJB2-109-1177-s001.docx]

**Appendix S5**: Cross-validation error results from ADMIXTURE runs from K = 1 – 10 for A) *C. sativa* and 2n = 40 *C. microcarpa* and B) 2n = 38 *C. microcarpa* datasets.
