# Supplementary material for: Molecular and archaeological evidence on the geographical origin of domestication for Camelina sativa
Source: Am J Bot. 2022 Jul 11;109(7):1177–90. doi: 10.1002/ajb2.16027 (PMC9542853; doi:10.1002/ajb2.16027)
Supplement: Supplementary file 6 — Appendix S6. Supplementary references for archaeological literature survey. References provided here correspond to those cited in Appendix S7 and comprise the body of literature used to determine the geographic scope of Camelina archaeological findings and their timing and cultivation status. [file AJB2-109-1177-s005.docx]

**Appendix S6**: Supplementary references for archaeological literature survey. References provided here correspond to those cited in Appendix S7 and comprise the body of literature used to determine the geographic scope of Camelina archaeological findings and their timing and cultivation status.

Andresen, S. T., and S. Karg. 2011. Retting pits for textile fibre plants at Danish prehistoric sites dated
between 800 BC and AD 1050. *Vegetation History and Archaeobotany* 20: 517–526.

Andrikopoulou-Strack, J.-N., P. Enzenberger, K. Frank, C. Keller, and N. Klän. 1999. Eine frührömische Siedlung in Jüchen-Neuholz. Überlegungen zur Siedlungskontinuität in der Lößbörde. *Bonner Jahrbücher*: 141–180.

Bakels, C. C., S. Arnoldussen, E. A. G. Ball, J. van Dijk, and E. V. N. Norde. 2019. Een ijzertijdboer zaait huttentut (*Camelina sativa*). *Metaaltijden 6*: 185–191.

Bouby, L. 1998. Two early finds of gold-of-pleasure (*Camelina* sp.) in middle Neolithic and Chalcolithic sites in western France. *Antiquity* 72: 391–398.

Brinkkemper, O., and R. de Man. 1999. Botanishce macroresten. *Castricum-Oosterbuurt, bewoningssporen uit de Romeinse tijd en middeleuwen*: 161–170.

Çizer, Ö. 2015. Archaeobotanical investigations of plant cultivation and husbandry practices at the Early Bronze Age settlement Küllüoba in West-Central Turkey: Considerations on environment, climate and economy. British Archaeological Reports Limited.

Dönmez, E. O., and O. Belli. 2007. Urartian plant cultivation at Yoncatepe (Van), eastern Turkey. *Economic Botany* 61: 290.

Engelhardt, B., F. Herzig, Z. Kobyliński, D. Krasnodębski, M. Kupryjanowicz, and M. Michniewicz. 1999. Early Medieval wells from Pfettrach, Bavaria: cultural ecology of an early Bavarian village. *Archaeologia Polona* 37: 87–118.

Gyulai, F. 2010. Archaeobotany in Hungary. *Seed, fruit, food and beverages remains in the Carpathian basin: an archaeobotanical investigation of plant cultivation and ecology from the Neolithic until the late Middle Ages. Archaeolingua, Budapest*.

Hansson, A.-M., K. Lidén, and S. Isaksson. 1993. The charred seed-cake from Eketorp. *PACT (Rixensart)*: 303–315.

Henriksen, P. S., D. E. Robinson, and K. Kristina. 2018. Bronze Age agriculture, land use and vegetation at Bjerre Enge based on the results of archaeobotanical analyses. Bronze Age Settlement and Land-Use in Thy, Northwest Denmark, 387–458. Jysk Arkæologisk Selskab.

Hovsepyan, R., and G. Willcox. 2008. The earliest finds of cultivated plants in Armenia: evidence from charred remains and crop processing residues in pisé from the Neolithic settlements of Aratashen and Aknashen. *Vegetation History and Archaeobotany* 17: 63–71.

Jacomet, S., C. Jacquat, C. Maise, J. Schibler, B. Stopp, J. Studer, L. Wick, and M. Winter. 1999. Climat, environnement, économie agricole et alimentation. *Müller F., Kaenel G. & Lüscher G., La Suisse du Paléolithique à l’aube du Moyen-Age IV Age du Fer. SGUF, Bâle*: 9–1.

Karg, S. 2012. Oil-rich seeds from prehistoric contexts. *Acta Palaeobotanica* 52: 17–24.

Kroll, H. 2000. Agriculture and arboriculture in mainland Greece at the beginning of the first millennium BC. *Pallas*: 61–68.

Kroll, H. 1991. Südosteuropa. *Progress in old world palaeoethnobotany. Balkema, Rotterdam*: 161–177.

Kucan, D. 2007. Archäobotanische Untersuchungen zu Umwelt und Landwirtschaft jungbronzezeitlicher Flussmarschbewohner der Siedlung Rodenkirchen-Hahnenknooper Mühle. *Ldkr. Wesermarsch. Probleme der Küstenforschung im südlichen Nordseegebiet* 31: 17–83.

Larsson, M. 2013. Cultivation and processing of *Linum usitatissimum* and *Camelina sativa* in southern Scandinavia during the Roman Iron Age. *Vegetation History and Archaeobotany* 22: 509–520.

Latałowa, M., 1998. Botanical analysis of a bundle of flax (Linum usitatissimum L.) from an early medieval site in northern Poland; a contribution to the history of flax cultivation and its field weeds. *Vegetation History and Archaeobotany* 7: 97–107.

Märkle, T. 2000. Die Wildpflanzen der Cortaillod-moyen-zeitlichen Besiedlung von Concise-sous-Colachoz, Kt. Waadt, Schweiz. *Archäologische Informationen* 23: 277–280.

Marston, J. M. & Castellano, L. 2021. Archaeobotany in Anatolia. in The Archaeology of Anatolia, Volume IV: Recent discoveries (2018-2020). eds. Steadman, S. R. & McMahon, G. 338–354 Cambridge Scholars Publishing.

Marston, J. M. 2017. Agricultural sustainability and environmental change at ancient Gordion. University of Pennsylvania Press.

Marston, J. M., and N. F. Miller. 2014. Intensive agriculture and land use at Roman Gordion, central Turkey. *Vegetation History and Archaeobotany* 23: 761–773.

Matney, T., T. Greenfield, K. Köroğlu, J. MacGinnis, L. Proctor, M. Rosenzweig, and D. Wicke. 2015. Excavations at Ziyaret Tepe, Diyarbakir Province, Turkey, 2011-2014 Seasons. *Anatolica* 41: 125–176.

Miller, N. F. 2011. Botanical aspects of environment and economy at Gordion, Turkey. University of Pennsylvania Press.

Nesbitt, M. 1996. Chalcolithic crops from Kuruçay Höyük: an interim report. *Kuruçay Höyük II. Türk Tarih Kurumu, Ankara*: 89–93.

Reed, K. 2015. From the field to the hearth: plant remains from Neolithic Croatia (ca. 6000–4000 cal bc). *Vegetation history and archaeobotany* 24: 601–619.

Riehl, S. 1999. Bronze Age environment and economy in the Troad: BioArchaeologica.

Riehl, S., M. Benz, N. J. Conard, H. Darabi, K. Deckers, H. F. Nashli, and M. Zeidi-Kulehparcheh. 2012. Plant use in three Pre-Pottery Neolithic sites of the northern and eastern Fertile Crescent: a preliminary report. *Vegetation History and Archaeobotany* 21: 95–106.

Rivera, D. N., G. M. Séiquer, C. O. de Castro, and F. J. A. Ariza. 2011. Plants and Humans in the Near East and the Caucasus: Ancient and Traditional Uses of Plants as Food and Medicine: a Diachronic Ethnobotanical Review:(Armenia, Azerbaijan, Georgia, Iran, Iraq, Lebanon, Syria, and Turkey). edit. um, Ediciones de la Universidad de Murcia.

Rosenzweig, M. S. 2014. Imperial environments: The politics of agricultural practice at Ziyaret Tepe, Turkey in the first millennium BCE. The University of Chicago.

Runge, M., and P. S. Henriksen. 2007. Danmarks ældste hørindustri. *Fynske minder* 2007: 145–168.

Schlichtherle, H. 1978. Vorläufiger Bericht über die archäobotanischen Untersuchungen am Demircihüyük Nordwestanatolien.

Stika, H.-P., and A. G. Heiss. 2013. Plant cultivation in the Bronze Age. The Oxford Handbook of the European Bronze Age.

Stroud, E. 2016. An archaeobotanical investigation into the Chalcolithic economy and social organisation of central Anatolia. PhD Thesis. University of Oxford.

Summers, J. 2020. *Analysis of bulk samples from land at Broadland Gate, Postwick, Norfolk (ENF147068, P8279)*. Archaeological Solutions Ltd.

Toulemonde, F., L. Bouby, P. Marinval, V. Zech-Matterne, A. Bouchette, M. Cabanis, M.-F. Dietsch-Sellami, et al. 2010. *Camelina sativa*: l’or végétal du Bronze et du Fer. *Anthropobotanica*.

Tumanyan, M. G. 1944. Crop plants of Urartu period in Armenian SSR. *Notices of AS of USSR, Social sciences*: 1–2.

Wasylikowa, K., M. Carciumaru, E. Hajnalova, B. P. Hartyanyi, G. A. Pashkevich, and Z. V. Yanushevich. 1991. East-Central Europe. *Progress in old world palaeoethnobotany*: 207–239.

Wiethold, J. 1999. Pflanzenreste des Mittelalters und der frühen Neuzeit aus zwei Kloaken in der Hansestadt Rostock. *Bodendenkmalpflege Mecklenburg-Vorpommern, Jahrbuch* 46: 409–432.

van Zeist, W., and J. H. Bakker-Heeres. 1985. Archaeobotanical studies in the Levant. 4. Bronze Age sites on the north Syrian Euphrates. *Palaeohistoria* 27: 247–316.

Zohary, D. Hopf, M. 1993. Domestication of plants in the old World-The origin and spread of cultivated plants in West Asia, Europe, and the Nile Valley. No. Ed. 2. Oxford university press.
