# Supplementary material for: Molecular and archaeological evidence on the geographical origin of domestication for Camelina sativa
Source: Am J Bot. 2022 Jul 11;109(7):1177–90. doi: 10.1002/ajb2.16027 (PMC9542853; doi:10.1002/ajb2.16027)
Supplement: Supplementary file 8 — Appendix S8. Analysis of final SNP data set for 60 individuals clustering with the 2n = 38 C. microcarpa group. (A) PCA, colored dots represent individual's country of origin. (B) Admixture plot at K = 2; USA and Europe genetic group, pink; S. Ukraine genetic group, brown. [file AJB2-109-1177-s006.docx]

**Appendix S8**: Analysis of final SNP dataset for 60 individuals clustering with the 2n = 38 *C. microcarpa* group. A) PCA, colored dots represent individual’s country of origin. B) Admixture plot at K = 2; USA and Europe genetic group, pink; S. Ukraine genetic group, brown.
