# Supplementary material for: Molecular and archaeological evidence on the geographical origin of domestication for Camelina sativa
Source: Am J Bot. 2022 Jul 11;109(7):1177–90. doi: 10.1002/ajb2.16027 (PMC9542853; doi:10.1002/ajb2.16027)
Supplement: Supplementary file 9 — Appendix S9. Mapped locations of 2n = 38 C. microcarpa, pink circles with black centroid and 2n = 40 C. microcarpa, blue circles, from (A) United States and (B) Europe and the Caucasus. USDA GRIN accessions from Europe were approximately mapped due to missing coordinate data. [file AJB2-109-1177-s009.docx]

**Appendix S9**: Mapped locations of 2n = 38 *C. microcarpa*, pink circles with black centroid and 2n = 40 *C. microcarpa*, blue circles, from A) United States and B) Europe and the Caucasus. USDA GRIN accessions from Europe were approximately mapped due to missing co-ordinate data.
